# An econometric approach to aggregating multiple cardiovascular outcomes in German hospitals

**DOI:** 10.1007/s10198-022-01509-y

**Published:** 2022-09-16

**Authors:** Angela Meggiolaro, Carl Rudolf Blankart, Tom Stargardt, Jonas Schreyögg

**Affiliations:** 1 Hamburg Center for Health Economics, Universität Hamburg, Hamburg, Germany; 2 KPM Center for Public Management, Universität Bern, Bern, Switzerland; 3 Swiss Institute for Translational and Entrepreneurial Medicine (sitem-insel), Bern, Switzerland

**Keywords:** Quality of care, Hospital, Accelerated failure time model, Cardiovascular diseases, I11

## Abstract

**Objective:**

Development of an aggregate quality index to evaluate hospital performance in cardiovascular events treatment.

**Methods:**

We applied a two-stage regression approach using an accelerated failure time model based on variance weights to estimate hospital quality over four cardiovascular interventions: elective coronary bypass graft, elective cardiac resynchronization therapy, and emergency treatment for acute myocardial infarction. Mortality and readmissions were used as outcomes. For the estimation we used data from a statutory health insurer in Germany from 2005 to 2016.

**Results:**

The precision-based weights calculated in the first stage were higher for mortality than for readmissions. In general, teaching hospitals performed better in our ranking of hospital quality compared to non-teaching hospitals, as did private not-for-profit hospitals compared to hospitals with public or private for-profit ownership.

**Discussion:**

The proposed approach is a new method to aggregate single hospital quality outcomes using objective, precision-based weights. Likelihood-based accelerated failure time models make use of existing data more efficiently compared to widely used models relying on dichotomized data. The main advantage of the variance-based weights approach is that the extent to which an indicator contributes to the aggregate index depends on the amount of its variance.

## Introduction

Quality of care can be defined as health improvement measured against the best possible outcome that can be achieved by applying current medical knowledge [1]. Previous research on evaluating quality of care has shown that health outcomes appraisals stem from factors at the individual level (e.g. age, clinical conditions, comorbidities); the health care provider level, which includes hospitals (e.g., health technology provision, staff organization); and the socio-cultural level (e.g., social policies, access to services) [2–4]. Information about the quality of care in health care institutions is widely used in the health economics literature, as well as in health policy decision making. In most cases, this information comprises (risk-adjusted) mortality rates or derivatives thereof, such as the difference between actual and predicted mortality rates [5, 6]. Given that the overall mortality estimate—and in-hospital mortality, in particular—is considered an approximation for hospital quality [7], more recent research has focused on hospital-level mortality that is specific to individual conditions. Additionally, most studies evaluating hospital quality have used only one quality indicator, despite the fact that researchers agree that quality is a multidimensional construct [8]. Moreover, even those studies that have considered more than one quality measure have commonly evaluated each measure separately [9, 10].

The main aim of this study was to develop and validate an aggregate index to assess the quality of hospital care in Germany and to observe how the variability in hospitals’ performance was distributed across the hospitals in our sample. In general, hospitals may differ in terms of structural factors, material resources, ownership and organization [11]. Although the actual associations between-hospital characteristics and quality of care are still debated in the scientific literature [12, 13], numerous studies have documented disparities in the quality of care for CVD that cannot be explained merely by differences in clinical factors [14, 15]. In this light, the European Society of Cardiology guidelines recommend a broader approach to measuring quality of care for CVD that includes health care providers and clinical outcomes evaluation [15]. Moreover, comparing the quality of hospital care across countries allows policy makers and clinicians to identify specific areas for improvement. However, despite the growing scientific interest in composite indices, policy makers have been reluctant to adopt them because of concerns about their reliability [16, 17].

## Methods

To assess the quantity of stochastic variation attributable to hospitals, a composite index requires: (a) different dimensions of quality that can be measured with distinct indicators, (b) intervention-specific risk adjustment to control for hospital variability, and (c) aggregation of different dimensions of quality. Our proposed methodology uses likelihood-based accelerated failure time (AFT) models and controls for correlation across different outcome measures by a simultaneous equations estimation. The variance-based weights aggregate different outcome measures into a single quality index. The advantage of the aggregation based on statistical weights is two-fold: first, the variance weights enable and address the precision of the measurement. Each specific outcome measure contributes more to the aggregate quality index when it has less variance because we divide the contribution to the overall outcome estimate by the respective inverse. Furthermore, the weights reduce the effects of heteroscedasticity.

Second, data-driven weights are robust to normative decisions [18–20]. A viable two-stage procedure to derive a quality indicator for hospital quality was suggested by Chua et al. [19]. In the same fashion, we aggregated two quality outcomes—mortality and readmissions—across four types of cardiovascular intervention by controlling for distinct intervention-specific risks. In the first stage, we risk-adjusted mortality and readmissions through a system of AFT simultaneous regressions and computed the weights based on the variance from the first stage. In the second stage, we derived the hospital fixed effect estimates through a weighted AFT model. Unlike the linear regression adopted by Chua et al., the AFT model included two quality outcomes and accommodated time as endogenous parameter. Finally, we ranked the hospital estimates into league tables based on the aggregate index. In particular, we observed how hospital ownership affected the resulting ranking.

### Setting

As a setting for developing and validating an aggregate index we used the German hospital market. In Germany, patients can choose between almost 2000 hospitals, which provide almost 500,000 hospital beds making Germany one of the countries with the highest number of beds per inhabitant in the OECD [21]. Hospitals belong to three ownership types: Public hospitals, private not-for-profit hospitals and private for-profit hospitals. These three types coexist, but with different market shares. All ownership types can be equally chosen by patients independently from their insurance status. Hospital reimbursement is mainly based on DRGs which are not linked to quality of care. Geographical variations within Germany may arise from the fact that hospitals located in eastern or western Germany are different in terms of their infrastructure. After German reunification in 1990, hospitals in East Germany (former GDR) received comparably higher subsidies from the federal government to upgrade their infrastructure. However, the east–west convergence in mortality for chronic ischemic heart diseases was slow until the early 2000s, probably attributable to the initial historical disadvantage among Germans from former East Germany [22]. In recent years, Germany introduced several policies to improve quality of care and increase quality transparency to help patients make informed choices. For example, it introduced mandatory, nationwide quality reports (§136b SGB V) and some sickness funds and other initiatives measured and published hospital quality (e.g., Wissenschaftliche Institut der Ortskrankenkassen (WIdO) [23]). So far, all initiatives measured quality based on single indicators and not based on aggregate indices.

### Data and patient selection

For our analysis, we used patient-level administrative data from BARMER, a large statutory health insurer in Germany with almost nine million members. In addition to general patient characteristics, such as age and sex, our data set included information on patients’ length of stay, inpatient and outpatient diagnoses according to the International Classification of Diseases (ICD-10), procedure codes according to the German Procedure Classification (OPS) system, and an identifier for the admitting hospital. Our analysis spanned a period of 12 years, from 2005 to 2016.

We used data from individuals aged 20 years or older who underwent, for the first time, one of the following four types of clinical intervention: (1) elective coronary artery bypass graft (CABG), (2) elective cardiac resynchronization therapy using an implantable pacemaker or implantable cardioverter-defibrillator (ICD/CRT), (3) hospital emergency treatment for ST elevation myocardial infarction (STEMI), or (4) hospital emergency treatment for non-ST elevation myocardial infarction (NSTEMI). We coded elective CABG (5–361 and 5–362) and elective ICD/CRT (5–377) according to the German procedure codes for surgery. We identified the two types of acute myocardial infarction (i.e., STEMI and NSTEMI) by hospital diagnosis codes (ICD-10). We categorized patients who received a primary diagnosis of I21.0-I21.3 (i.e., an acute transmural myocardial infarction) as STEMI patients and those who received a primary diagnosis of I21.4 (i.e., acute sub endocardial myocardial infarctions) as NSTEMI patients. Patients who underwent revascularization through CABG or ICD/CRT after AMI were categorized as STEMI and NSTEMI emergency treatment patients, because the AMI was considered to be the triggering event in these cases [15, 24]. Patients who could not be assigned to a distinct group were excluded from the analysis (e.g., patients who underwent CABG and ICD/CRT during the same hospitalization episode).

To identify the episode of care during which patients underwent one of the four interventions for the first time, we excluded individuals who had been hospitalized or who were not observable during the two years (i.e., 730 days) immediately preceding the intervention. Furthermore, we excluded episodes in which patients were transferred to another hospital during their stay because we could not clearly attribute outcomes to a specific hospital in such cases. We also excluded hospitals with fewer than 20 records for any of the four intervention types because the results would not be statistically reliable.

At the hospital level, we included the following variables: hospital ownership (public, private for-profit, and private not-for-profit hospitals), hospital teaching status, staff (number of physicians and professional nurses)*,* hospital capacity (number of beds)*,* volume (number of admissions and discharges per year and ward), location (eastern or western Germany) [24, 25]. The last of these may also reflect spillover effects from German reunification [22]. In addition, we included gross regional product (GRP) per capita as a proxy for inequality in health outcomes, extracting data from the German INKAR database [26, 27].

### Quality outcomes

For each of the four types of intervention, we used two outcomes—mortality and readmission—as proxies for hospital quality. We defined mortality as the number of days between first admission to hospital and death, and we defined readmission as the time between discharge and readmission. We considered an event to be a readmission only if the new stay was related to the initial admission, as indicated by a diagnosis code for a heart-specific disease (I20–I25, excluding I25.2, and I30–32, I34–I38, I44–I50, and I51.2), cardiogenic shock (R57), complications of cardiac and vascular prosthetic devices, implants, and grafts (T82), or other functional disturbances following cardiac surgery (I97.1). Furthermore, we adjusted for severity of readmission using the total length of stay after the first admission. Therefore, we computed standardized days to readmission by dividing the raw days to readmission by the total standardized days spent in the hospital.

The follow-up period was set at 365 days to balance between the increasing effects of post-discharge care, for which hospitals are not responsible, and the loss of information that would result from using a shorter period [5]. Individuals who either survived beyond, or were not readmitted within, 365 days after the event were considered to be right-censored observations. To account for the competing risks of death and readmission, observations were coded as censored-readmission in case of death [28].

### Model specification

The main statistical challenge in setting an aggregate index (*Q*) is to summarize multiple measures of quality performance into a unidimensional-aggregated score:

1 $${Q}_{h}={\sum }^{k}{W}_{k}{X}_{kh},$$

where *k* is the matrix of intervention-specific outcomes, *W*_*k*_ the weight attached to *k*, and *X*_*kh*_ the score *k*-specific attributed to hospital *h* [18, 29–31]. To address the uncertainty surrounding a composite outcome measure, we adopted the variance weights approach. We employed a two-stage approach to measure hospital quality on the aggregate level. The first stage of the AFT model produced estimates for hospitals along with the respective outcome and intervention*.* In the second stage, we estimated and aggregated the quality outcome measures using the weights computed in the first stage. Afterwards we inserted the hospital dummies into the second step regression, normalized the results into an index ranging from zero to 10, and ranked the hospital estimates and respective confidence intervals. Finally, we examined how hospitals performed on our quality index according to hospital ownership.

#### First-stage estimation

In the first stage, we simultaneously estimated eight equations (i.e., one equation for each of the two quality outcomes and each of the four types of intervention). We modeled time to event (i.e., readmission or death) as a function of the patient characteristics of individuals*,* outcomes*,* interventions, hospitals using an AFT model (Eq. 2). Individual patient characteristics were denoted by matrix *X*, outcome- and intervention-specific dummies by matrix *V*, outcome- and intervention-specific hospital covariates by matrix Z, and macroeconomics and geographical regional characteristics by matrix $\Gamma$. *β* represents a vector of the patient characteristics, *λ* describes a vector of outcome- and intervention-specific parameter estimates, and *γ* is a vector describing outcome- and intervention-specific hospital characteristics. To represent hospital competition, we included the vector *δ*, describing the GRP per capita and the geographical location of the hospital (i.e., one of the 16 German states) (full specifications of Eq. 2 in Appendix A1).

2 $$\mathrm{ln}\left(T\right)=X\beta +V\lambda +Z\gamma +\Gamma \delta +\varepsilon .$$

Overall, we identified three levels of cross variation: patients, hospitals, and regions. The error terms capture variation in-hospital quality performance after allowing for differences in patient characteristics and hospital characteristics. Patient characteristics were age, age-squared, sex (female = 1), comorbidities and the distance between place of residence and admitting hospital (for STEMI and NSTEMI only). We explicitly measured comorbidity before admission based on data from non-hospital outpatient care (i.e., we did not consider comorbidity at admission) because diagnosis codes that are registered during the hospital episode might be prone to manipulation [32]. We coded patient comorbidities using two different scales depending on the indication. For ICD/CRT and CABG, we used the 31 Elixhauser comorbidity groups [33, 34] and to control for comorbidities in cases of STEMI and NSTEMI, we used the nine AMI-specific Ontario mortality prediction rules [3]. In both cases, we coded the comorbidity groups using dummy variables.

For STEMI and NSTEMI, we used the distance between patients’ place of residence and their admitting hospital as a proxy for the time elapsed between the acute event and hospital admission as immediate action after an AMI has a significant impact on mortality [35]. We calculated the distance between the center of the patients’ home postal code area and the admitting hospital using the orthodromic distance. To reduce confounding from a potential correlation between hospitals and patient-level attributes, we included hospital covariates, i.e., volume, staff, capacity, ownership, and teaching affiliation. We expressed volume as the number of admissions and discharges per year for each hospital ward. The hospital staff was classified into the number of specialist physicians and professional nurses and hospital capacity was expressed as the number of beds. For ownership, we distinguished between private for-profit, private not-for-profit, and public; furthermore, we added the hospital teaching status. To disentangle a possible joint contribution of ownership and hospital capacity to quality outcomes [28, 36, 37], we included interactions between-hospital ownership and the respective number of beds. We considered GRP per capita as well as Eastern and Western Germany as region-level covariates.

#### Outcome-specific weights and aggregation in second-stage regression

Weights in the second stage were based on the precision of the estimation of the specific outcomes in the first stage. The main assumption of the variance-weighted regression is that if we can predict an outcome more precisely, the weights of the corresponding observations will be higher. In the second stage, we used the AFT model to examine hospitals as driving factors of variation in quality outcomes. We again simultaneously estimated mortality and readmissions for each of the four interventions as a function of individual patient characteristics, hospital and regional covariates, and, lastly, hospital identifiers (Eq. 3). In doing so, we adapted the model from the first stage in two ways. First, we weighted each observation with its outcome- and intervention-specific weight and, second, we included a matrix *R* instead of a matrix *Z*. Matrix *Z* (Eq. 1) consists of outcome- and intervention-specific hospital fixed effects*,* so *R* (Eq. 3) consists of hospital ID dummies and represents the hospital-specific fixed effect because, in this stage, conditions were aggregated into one index. Therefore, *ρ* is a vector of *h* aggregated hospital estimates, i.e., the vector *ρ* reflects unstandardized aggregate hospital quality (full specification of Eq. 3 in Appendix A2).

3 $$\mathrm{ln}\left(T\right)=\mathrm{R\rho }+X\beta +V\lambda +\Gamma \delta +\varepsilon .$$

We interpreted the hospital estimates and the confidence intervals in Eq. (3) as a measure of relative hospital quality, expunged of the effect of case mix and hospital characteristics. For readability, we standardized the aggregated hospital quality parameter *ρ* estimates and the corresponding confidence intervals into a scale ranging from zero to 10.

### Sensitivity analysis

To test for internal validity, we set several scenarios and simulated results. First, we increased the survival time for all uncensored patients from 45 randomly selected hospitals (approximately 10% of the sample). We simulated values for a (1) 30%, (2) 50%, and (3) 100% increase in time to death. Because increased survival represented a better outcome, we hypothesized that the ranking of the manipulated hospitals should increase.

Second, we checked whether our risk adjustment was appropriately implemented. We used the same set of 45 hospitals and simulated an increase in comorbidity by randomly increasing the Elixhauser comorbidity score at the patient level by 50%. Again, we hypothesized that the ranking of the manipulated hospitals should increase. For both sets of scenarios, we used single-sided non-parametric Wilcoxon signed-rank tests to check the significance of any improvement in quality ranking.

Third, we analyzed whether aggregation leads to unexpected results. Therefore, we calculated a separate index for each of the four interventions and analyzed the correlation between each of these and the aggregated index. To do so, we restricted the sample in the first stage to one of the four interventions and determined the weights for the two outcomes (i.e., mortality and readmission). In the second stage, we aggregated the results to obtain an intervention-specific quality index. We expected correlation between the ranks of the intervention-specific indices and those obtained using the fully aggregated index, as well as larger confidence intervals because the intervention-specific indices rely on fewer observations.

## Results

### Descriptive results

We identified 175,574 episodes that fulfilled our inclusion criteria. The episodes occurred in 452 hospitals and could be categorized into 23,735 CABG, 55,415 ICD/CRT, 37,007 STEMI and 59,417 NSTEMI patients. Overall, 10,876 patients died, and 157,046 were readmitted. In total, 164,698 observations were right-censored due to death and 74,787 due to readmission. Patient characteristics by intervention are presented in Table 1.

**Table 1:** General patient characteristics by intervention type

	CABG	ICD/CRT	STEMI emergency treatment	NSTEMI emergency treatment
*Observations*	23,735	55,415	37,007	59,417
Uncensored (outcome = death)	142	646	1,937	8,151
Uncensored (outcome = readmission)	22,291	51,995	32,372	50,388
*Demographics*				
Age [years (STD)]	68.2 (8.8)	70 (12.4)	62.4 (11.8)	67.6 (12.3)
Sex (female = 1) [%]	25.5	49.3	31	41
Distance to hospital [km]	22.6 (19.5)	11.9 (13.7)	11.53 (12.4)	9.89 (11.2)

See Appendix A3 for Elixhauser Comorbidity groups and Ontario myocardial infarction prediction rules

On average, the Elixhauser score was higher for teaching or public hospitals (14.93 and 14.86, respectively) than for private for-profit or private not-for-profit hospitals (14.39 and 14.6, respectively). In our sample, public hospitals had 925 beds (SD 567), private for-profit hospitals 490 beds (SD 340), and private not-for-profit hospitals 441 beds (SD 248) on average. In total, 13.7% of the public hospitals were teaching hospitals, as well 28% of those that were private for-profit and 19% of those that were private not-for-profit. Overall, public hospitals employed the highest number of specialist doctors (222, SD 196), and private not-for-profit the lowest (79, SD 66). The mean number of nurses employed in public hospitals was 722 (SD 595), whereas in private not-for-profit hospitals it was 288 (SD 180) and in private for-profit hospitals 349 (SD 235). On average, the number of discharges per year was higher for private for-profit hospitals (i.e., 822, SD 930) than it was for public hospitals (625, SD 847) or for private not-for-profit hospitals (428, SD 651). Annual admissions to cardiology departments were higher for private for-profit hospitals, which had a mean of 930 (SD 1,120) admissions compared to 835 (SD 1,146) in public and 613 (SD 972) in private not-for-profit hospitals.

### Regression results

The results of the first- and second-stage regressions are shown in Appendix A4–A5. The case-mix covariates are commented in Appendix A5. We observed a total of eight outcome measures for each hospital (i.e., two for each of the four intervention types) and used their variances as weights in stage two. In the second AFT regression, we kept hospital covariates and included hospital dummies. We interpreted the resulting coefficients as aggregated mortality and readmission odds and therefore as a measure of hospital quality [23]. Overall, comorbidities that were significant in the first AFT step remained significant in the second AFT regression. After weighting, distance from hospital in cases of AMI was no longer significant, whereas private ownership became significant.

The hospital coefficients had smaller standard errors (SE) in the weighted AFT, which indicates that risk-adjustment substantially reduced variation in the raw hospital estimates. Among the covariates representing hospital capacity, only the interaction between the covariates for the number of beds and teaching-hospital status was significant, with a negative coefficient; each extra bed was associated with a one percent decrease in expected time to death or readmission. Regarding hospital ownership, the hospital dummy for private for-profit hospitals produced a significant coefficient (*p* = 0.0144) with a negative sign (ODDS = 0.84). Hence, for hospitals of this ownership type, the time to readmission or death was 16% shorter than other hospital ownership types. The hospitals located in western Germany (*p* = 0.0075) appeared to have an expected time to event that was 57% shorter than that for hospitals located in eastern Germany (ODDS = 0.43). The covariate for hospital volume, which was expressed as admissions per year, was significant but the coefficient was nearly zero. The yearly GRP per capita was significant; however, the negative coefficient was close to zero.

### Index plot and league tables

The precision-based weights were higher for mortality than for readmission in all cases. Regarding mortality, ICD/CRT produced the largest variance and thus the smallest weight, whereas CABG and STEMI produced the smallest variance and the greatest weights. As expected, the model captured mortality with greater precision than it did with readmissions despite the former comprising a much smaller number of observations than the latter (i.e., 10,876 vs 157,046). Regarding mortality, CABG and STEMI had almost the same weights (142 and 1937 observations respectively). STEMI had a higher weight than NSTEMI (8151 observations). Probably the model captured the short-term effects of STEMI with greater precision.

Mortality attributable to cardiac surgery has declined dramatically over time. Nevertheless, isolating the effect of CABG and ICD/CRT on survival from the underlying heart condition was not straightforward. We observed only 142 cases of CABG, and the weight assigned after the first-stage regression had low variance. In AMI treatment, new revascularization technologies, such as percutaneous coronary intervention (PCI), are now favored over CABG, which today is mostly performed to relieve symptoms in selected patients who are unresponsive to standard medical treatment [38]. ICD/CRT had the largest variance and the lowest weight. Non-cardiac comorbidities or unobserved clinical factors may contribute to an increased risk of death following ICD implantation [39].

Concerning readmissions after hospitalization for AMI, NSTEMI and STEMI had the lowest weights, despite a large number of observations (50,388 and 32,372, respectively). ICD/CRT readmission had the highest weight value, which was consistent with this outcome having the greatest number of observations (51,995) and a small variance. Regarding bypass, 87.3% of readmissions generally occur for reasons related to CABG itself [40], and are mostly associated with the risk factors that we controlled for, and indeed the variance we obtained was small (Table 2).

**Table 2:** Aggregation weights by outcome and intervention

Intervention	Weights	
	Mortality	Readmission
CABG	1.938	1.57
ICD/CRT	1.81	1.58
STEMI	1.936	1.53
NSTEMI	1.88	1.22

**Fig. 1:**
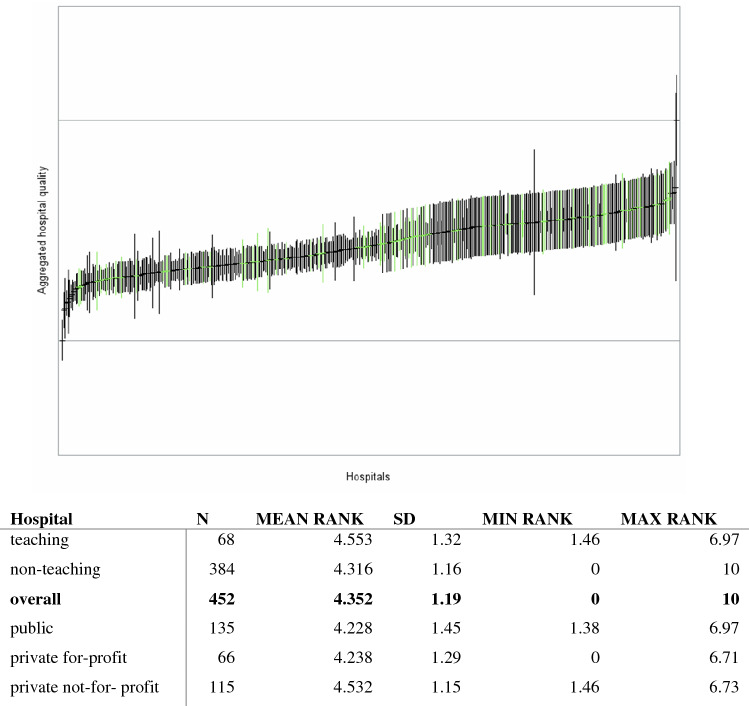
Aggregate hospital quality index and league table with ranking. Index range from 0 to 10

**Fig. 2:**
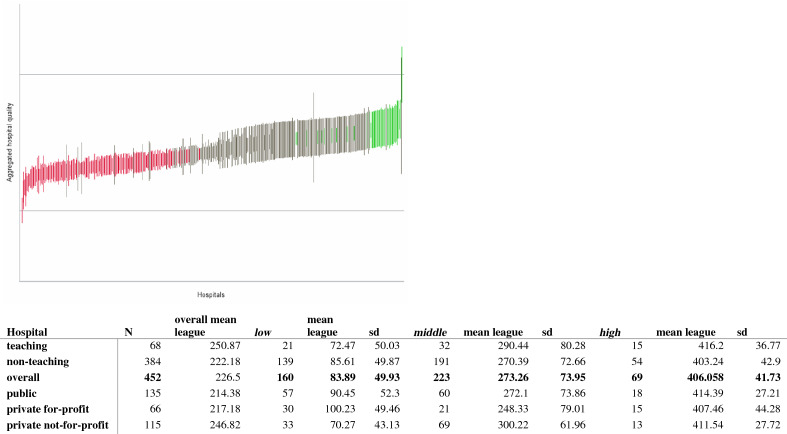
*Aggregate* hospital quality index and league table with hospital rankings: low = low performance (red); middle = middle performance (grey); high = high performance (green)

Figures 1 and 2 present the results of the standardized hospital parameter estimates and the corresponding confidence intervals. Hospitals providing a poorer service (i.e., resulting in earlier death or earlier/more severe readmissions) were ranked lower than hospitals providing a better service. As expected, hospitals with fewer observations or events during the observation period tended to have larger confidence intervals. Based on the two-stage AFT estimates, we built a ranking of hospitals. The worst performing hospital was ranked one and the best was ranked 452. Overall, the mean rank of hospitals’ performance was 4.35 over a range of one to 10 (Fig. 1). Estimates for teaching hospitals (78/452) were higher in general than those for non-teaching hospitals (374/452), with mean rank values of 4.56 and 4.31, respectively.

All values less than or equal to the mean estimate denoted poor performance and were labeled as’low’; conversely, hospitals scoring greater than or equal to 4.35 were grouped into the higher performance league table, labeled as ‘high’. The ‘middle’ group comprises standard performing hospitals (Fig. 2). As expected, teaching hospitals performed well in the ranking, with an average value of 4.55. Among these hospitals, 22% (15/68) were ranked as ‘high’ compared to 14% (54/384) of non-teaching hospitals. However, the worst teaching-hospital performers represented 30% (21/68) of the bottom group, suggesting a high degree heterogeneity.

In terms of hospital ownership, private not-for-profit hospitals scored on average the highest mean estimate (4.532) and a league table rank of 246.82, compared to ranks of 217.18 for private for-profit hospitals and 214.38 for public hospitals. For 136 hospitals, ownership information was missing in our data (Fig. 1). However, private not-for-profit hospitals did not perform as well in terms of top ranking hospitals: only 11.3% (13/115) were ranked in the ‘high' group, compared to 22.7% (15/66) of private for-profit hospitals and 13.3% (18/135) of public hospitals. Nevertheless, there were proportionately fewer private not-for-profit hospitals among the bottom group: 28.7%, compared to 42% (57/135) of public hospitals and 45.5% of private for-profit (Fig. 2). All sensitivity analyses supported the robustness of the model.

## Discussion

The approach proposed in this paper provides a viable method for aggregating several dimensions of hospital quality using precision-based weights. It has a number of important advantages: for example, compared to the widely used generalized linear models, which rely on dichotomous data, the likelihood-based, accelerated failure time survival models are more efficient. Additionally, the ability to use administrative inpatient and outpatient data enables providers and payers to follow patients across all health care sectors both before and after hospital admission, and to do so at reasonable cost. Lastly, the method also addresses the issue of selection bias, a major obstacle to comparing hospital quality outcomes, by allowing risk adjustments to be made in an intervention-specific manner.

Previous approaches to measuring the quality of hospital care lacked the following features, which we aimed to address. First, non-aggregated outcome measures might be computationally demanding and therefore hard to interpret for stakeholders [41]. Health care institutions pursue multiple objectives, and quality represents a multifaceted entity per se. Undoubtedly, condensing measurements of different outputs into a single measure and using this to rank hospitals or health care systems has the advantage of supplying a “big picture” to both policy makers and consumers [18]. However, some researchers have argued that composite measures are limited in their ability to provide an inclusive and comprehensive evaluation of quality. Indeed, any organization can perform successfully according to one indicator and less well on another [17]. As we mentioned earlier, inefficiency may be latent in non-competitive markets. Constraints on health care expenditure require the pursuit of efficiency, and performance analysis may become a benchmark for policy makers to allocate budgets [16, 30]. Our two-stage weighted AFT model produced a composite index of hospital performance that specifies an indirect measure of quality. We assumed that residuals may embed factors other than performance variation, such as measurement error or omitted model variables. In computing the weights, we aggregated eight outcome estimates. The resulting inverse variance was both precision-based and importance-based [19, 30]. The literature acknowledges a debate about stakeholder preferences and the elicitation of weights. In particular, Gutacker and Street (2018) question the legitimacy of this statistical approach, particularly whether it aligns with economic welfare theory and, by extension, matches stakeholder preferences [17]. However, while there is no consensus over the best method to elicit weights, the variance-based model has several advantages. First, defining weights at the macro level can prevent the shortfalls of a merely normative approach [18, 19]. Yet, low-incidence phenomena such as mortality can be incorporated in the analysis. Ultimately, the data-driven weights are not computationally demanding and are potentially replicable either in different healthcare circumstances or at broader policy level [19].

Second, in general, quality indicators rely on hospital episode data, leading to bias because the comorbidities before the intervention cannot be reliably determined. A relevant drawback of studies that rely solely on hospital data is the potential bias related to losses to follow-up [42]. In contrast, using administrative data from sickness funds allows the patient to be followed across all healthcare sectors for the periods before and after they are admitted to the hospital. In this way, we can capture both the health status and the outcome over the entire continuum of care. More importantly, inpatient diagnoses need to be screened for plausibility because they are highly relevant for reimbursement purposes and quality assessment, and therefore susceptible to upcoding [32]. Yet, the use of administrative data from sickness funds help minimizing this problem.

Third, accounting for selection bias is a major issue when comparing quality outcomes among hospitals because comorbidity confounds comparisons [19]. Basically, there are four sources of endogeneity: (a) healthier patients may select a hospital based on existing quality information, leading raw quality outcomes to be overestimated; (b) cream skimming: some hospitals may avoid patients who are at high risk of dying; (c) hospitals that are reimbursed on a case-by-case basis may focus on the most profitable (i.e., healthier) patients; and (d) hospitals in economically deprived regions might treat more patients with more comorbidities, because deprivation correlates with health status [43]. There are two basic strategies to account for this bias. First, one may focus on acute events that need immediate treatment, such as AMI. In all likelihood, patients will be admitted to the nearest and not to the best hospital in such circumstances. Second, the patient’s condition refers to the period before hospital admission [19]. Within the proposed model, we followed the second approach and accounted for patients’ comorbidities using two intervention-specific measures. We applied the 31 Elixhauser comorbidity groups for CABG and ICD/CRT; conversely, we used the Ontario AMI mortality prediction rules and the distance to hospital to account for comorbidities in case of STEMI and NSTEMI. By incorporating two different risk adjustments in our model, we accommodated the best sets of risk-adjustment variables for each intervention [44, 45]. At the same time, we attempted to control for deprivation and equity in access to care by considering hospitals’ geographic location and socio-economic variables at the regional level, such as GRP.

Fourth, several studies have used dichotomous data, such as in-hospital and/or 30-, 90-, and 180-day mortality, for quality assessment. This leads to a substantial loss of information compared to approaches that employ survival models [46]. Indeed, using likelihood-based survival models is the predominant choice for analyzing censored survival data because it efficiently uses all the information available [47]. Grouping different outcomes and interventions simultaneously allows for potential correlations among different outcome measures. Although we did not formally test for heteroscedasticity, we assumed correlation between different outcomes because of similar organizational structures within hospitals. For instance, if a single cardiovascular surgical team is responsible for all interventions this may cause correlations in outcome measures.

The literature offers a long list of internal and external drivers of hospital quality; however, any risk adjustment cannot capture the amount of persisting heterogeneity that we observed [48]. Because we extracted weights from the unexplained part of the model, hospital estimates indirectly quantify the extent of the distance from the mean performance estimate. Therefore, we could not provide new insights into the drivers of performance [49]. As expected, we encountered substantial heterogeneity among hospitals with higher performance. Overall, being a teaching hospital had a positive effect on quality compared to being a non-teaching hospital. Regarding the role of ownership in influencing hospital performance, the association was negative for private for-profit hospitals, whereas private not-for-profit hospitals had the strongest positive association. These findings are attributable only in part to the fact that patients referred to public hospitals generally have more severe conditions, whereas teaching hospitals are generally known to provide more specialized care. However, public hospitals in our sample tended to have standard performance and were scarcely represented at the bottom level. This might be related to the higher percentage of private for-profit and private not-for-profit hospitals in our sample that had teaching status. We were not able to quantify the extent to which the interaction between-hospital ownership and hospital beds capacity influenced the quality outcomes because the coefficients were not significant.

Concerning geographic location, the hospitals in western Germany had a lower performance than their Eastern German counterparts. This may be partly explained by the modern infrastructure of Eastern German hospitals due to large investments over the last decades. The lack of continuity between ambulatory care and hospital care particularly in West German regions might further contribute to a deterioration in patients’ condition and an increase in the risk of readmission and death [22].

Our study had several important limitations. Although administrative data contain information across all healthcare sectors over several years, such data lack clinical predictors that might add validity to the index [50]. For instance, we did not consider the time-to-balloon and the revascularization path for STEMI and NSTEMI [51]. Determining the mode of revascularization, whether this be a percutaneous coronary intervention (PCI) or a coronary artery bypass grafting (CABG), depends on patient-specific and anatomical considerations [51].

In theory, a patients’ health condition will probably improve or deteriorate over time. Although we fully exploited the dataset to reflect health status by considering primary and secondary inpatient and outpatient diagnosis codes, we did not have complete information on health status. Hence, for the time lapse between the first and second event, we considered the total length of stay as a proxy for severity.

Administrative data are generally consistent with patient chart data and typically correlate with clinical information. Aylin et al. [52] showed that the risk of dying can be similarly predicted by administrative and clinical databases [33, 52, 53]. Administrative data are also less prone to manipulation because they can capture the health status before the admission; this advantage clearly outweighs the lack of inpatient clinical data. However, the method is flexible enough to incorporate clinical data to improve the results, where available.

Although rare, mortality is clearly a valid outcome for cardiovascular diseases, but it has sometimes been considered inadequate because of measurement limitations and the fact that survival is not the primary aim in the context of palliative care or end-of-life treatment. Readmissions are potentially a robust alternative [34, 54] although they may occur for monitoring reasons. Therefore, we (1) considered only cardio-related admissions and (2) we weighted for the severity of patient condition by including the total length of hospital stay.

Letting data determine precision-based weights is transparent and cannot be influenced by normative decisions [19, 55]. The AFT weight estimates are overall consistent with the clinical literature. For instance, our model assigned a higher weight to STEMI, suggesting a more precise measurement. After revascularizations procedures, patients with STEMI have a worse short-term prognosis than patients with NSTEMI. However, NSTEMI patients in our sample had worse long-term survival after six months (9.0% for STEMI and 11.6% in NSTEMI) [15, 56]. The NSTEMI subset of AMI entails a widely varying risk of morbidity and mortality [51], explaining to some extent the low precision weight estimate with respect to STEMI [57]. Moreover, according to clinical practice guidelines, STEMI survival is time-sensitive because reperfusion therapy is the cornerstone of STEMI treatment [15]. By controlling for the distance from hospital, we considered this aspect only in part. Conversely, patients diagnosed with NSTEMI may be at higher risk of readmission than those initially admitted with STEMI for reasons that are still not fully understood. However, a risk of early readmission suggests the contribution of factors pertaining to transition of care that we cannot observe [58]. In our analysis, ICD/CRT readmission had the highest weight value. A large study found that 12% of patients who underwent cardiac electronic device implantation had an early readmission [59]. Finally, the use of precision-based weights does not preclude the implementation of additional, weights schemes, including patients’ willingness to pay or a policy-oriented valuation [19, 60, 61].

## Conclusion

In this study, we used a method based on variance weights to aggregate different quality outcomes across multiple interventions into an aggregate index of hospital quality. Our findings provide evidence that administrative data could be a valid source for informing and guiding decisions. Moreover, we observed how hospital ownership can contribute to variability in performance.

The proposed method provides a valid basis for assessing the quality of hospitals and its use may encourage—at least indirectly—quality-based competition in competitive hospital markets. Although empirical studies of the impact of hospital competition on the quality of health care have produced inconclusive and sometimes contradictory findings, some results suggest a positive impact on the quality of inpatient care [49, 55]. It is likely that these inconsistencies are due to different methodologies, hospital competition measures, and quality outcomes [10]. In imperfect healthcare markets, our index is not meant to support “carrot and stick” policies, but rather to be used for monitoring purposes. Nevertheless, the index may contribute to increase transparency when choosing a healthcare provider. For patients, a quality index based on statistical precision may represent an objective factor to consider when choosing among hospitals. However, other subjective factors, such as confidence in the referring physician, trust in the admitting healthcare hospital, proximity, or staff experience, will always influence patient decisions.

The index has the potential to be expanded to a broader range of interventions from different specialties and to healthcare organizations other than hospitals. As our example covers only a part of the actual cases in German hospitals, the between-hospital discriminatory power will increase if additional data from other health insurers become available. Finally, the methodological approach may be an important starting point for health economic and health services research that incorporates quality analyses.

## Data Availability

Not applicable.

## Appendix A1: Full specification of Eq. (2) (first stage of AFT model)

_1__*Nj*,1_)(_1,O__*Nj*,_.with j = 1…J intervention outcomes (where i = [1,… , I] corresponds to four interventions; o = [1, 0] to two outcomes [readmission, death]) and 1…N corresponding to the n^th^ patient level observation receiving the i^th^ intervention with outcome o. Tj = [(t1,1…t_N,J_)] corresponds to the occurrence of the j^th^ outcome for the n^th^ observation.

${X}_{j}^{c}$ = the element of matrix *X* describing [1,…, c] patient characteristics; Xj = [(X_1,1_…X_i,1_)…(X_1,0_…X_i,0_)] corresponds to the j^th^ =(i *x* o) intervention outcome.

*β* = the *c*’s element of vector *β* describing the parameter estimates of the patient’s characteristics.

${V}_{j}^{i x o}$ = the element of matrix *V* specified by dummy variables corresponding to [1,…,V] hospitals with the j^th^ specific outcome for the n^th^ observation.

${\lambda }_{i x o}$ = the *v*’s element of vector *λ* describes the outcome- and intervention-specific hospital dummies.

${Z}_{j}^{h}$ = the element of matrix *Z* describing the outcome- and intervention-specific [1,…, h] hospital characteristics of [1,….,V] hospitals; Z_J_ = [(Z_V,1x1_…Z_V,ix1_)…(Z_V,1x0_…Z_V,ix0_)] corresponds to the j^th^ intervention outcome in the v^th^ hospital for the n^th^ observation.

${\gamma }_{h}$ = the *h* element of vector *γ* that consists of parameter estimates describing outcome- and intervention-specific hospital characteristics.

${\Gamma }_{VJ}^{d}$ = the element of matrix $\Gamma$ describing regional-specific [1,…,d] characteristics for [1,…,V] hospitals located in d=[1,…,16] Regions; Γ_VJ_ = [(Γ_V,1x1_…Γ_V,ix1_)…(Γ_V,1x0_…Γ_V,ix1_)] corresponds to j^th^ in${\Gamma }_{V}$tervention outcome for the n^th^ observation in the v^th^ hospital located in the d^th^ Region.

${\delta }_{d}$ = the *d* element of vector *δ* consists  of parameter estimates describing Regional level characteristics for the v^th^ hospital located in the d^th^ Region.

${\varepsilon }_{j}$ = the element of vector ε that describes the normally distributed error term; ε_j _= [(ε_1,1_…ε_i,1_)…(ε_1,0_…ε_i,0_)] corresponds to the j^th^ intervention outcome.

## Appendix A2: Full specification of Eq. (6) (second stage of AFT model)

with j = 1…J intervention outcomes (where i = [1,…,I] corresponds to four interventions; o = [1,0] to two outcomes [readmission, death]) and 1…N corresponding to n = [1…N] patient level observations. Tj = [(t_1,1_…t_N,J_)] to the occurrence of the j^th^ outcome for the n^th^ observation.  The outcome- and intervention-specific weights W^J^ multiply the contribution to the log likelihood of  each observation.

${X}_{j}^{c}$ = the element of matrix *X* describing [1,…, c] patient characteristics; Xj = [(X_1,1_…X_i,1_)…(X_1,0_…X_i,0_)] corresponds to the j^th^ =(i *x* o) intervention outcome.

*β* = the *c*’s element of vector *β* describing the parameter estimates of the patient’s characteristics.

${V}_{j}$ = the element of matrix V specified by dummies corresponding to *1…V* hospitals.

${\varrho }_{V}$ =  the element of vector *ρ* that consists of parameter estimates describing the aggregated v^th^ hospital quality.

${Z}_{j}^{h}$ = the element of matrix *Z* describing the outcome- and intervention-specific [1,…, h] hospital characteristics of [1,….,V] hospitals; Z_VJ _= [(Z_V,1x1_…Z_V,ix1_)…(Z_V,1x0_…Z_V,ix0_)] corresponds to the j^th^ intervention outcome in the v^th^ hospital for the n^th^ observation.

${\gamma }_{h}$ = the  h’s element of vector *γ* that consists of parameter estimates describing outcome- and intervention-specific hospital characteristics.

${\Gamma }_{VJ}^{d}$ = the element of matrix $\Gamma$ describing regional-specific [1,…, d] characteristics for [1,…, V] hospitals located in d=[1,…, 16] Regions; Γ_VJ_ = [(Γ_V,1x1_…Γ_V,ix1_)…(Γ_V,1x0_…Γ_V,ix1_)] corresponds to j^th^intervention outcome for the n^th^ observation in the v^th^ hospital located in the d^th^ Region.

${\delta }_{d}$ = the *d* element of vector *δ* consists of parameter estimates describing Regional level characteristics for the v^th^ hospital located in the d^th^ Region. 

${\varepsilon }_{j}$ = the element of vector ε that describes the normally distributed error term; εj = [(ε_1,1_…ε_i,1_)…(ε_1,0_…ε_i,0_)] corresponds to the j^th^ intervention outcome. 

## Appendix A3: Prevalent comorbidities by intervention type

**Table:**

	CABG	ICD/CRT	STEMI emergency treatment	NSTEMI emergency treatment
**Elixhauser comorbidity groups [%]**				
Blood loss anemia	2.9	2.5		
Hypertension, complicated	41.4	39.23		
Hypertension, uncomplicated	89.1	82.06		
Chronic pulmonary disease	54.5	54.1		
Depression	43.2	46.76		
Drug abuse	1.9	2.72		
Weight loss	5.7	7.98		
Cardiac arrhythmia	57.7	86.37		
Valvular disease	53	45.32		
Congestive heart failure	52.3	53.23		
Psychoses	2.6	3.44		
Fluid and electrolyte disorders	13.7	16.38		
Hypothyroidism	22.5	27.22		
Metastatic cancer	4	4.3		
Solid tumor without metastasis	20.1	19.33		
Obesity	40.3	35.92		
AIDS/HIV	0.30	0.15		
Alcohol abuse	5.4	6.30		
Rheumatoid arthritis/collagen	20.2	22.52		
Diabetes, complicated	32.2	25.33		
Diabetes, uncomplicated	16.09	14,86		
Peripheral vascular disorders	57.76	40.5		
Coagulopathy	15.68	20.35		
Liver disease	17.97	29.31		
Pulmonary circulation disorders	8.15	11.77		
Lymphoma	2.16	2.24		
Paralysis	6.42	8.23		
Deficiency anemia	17.09	15.38		
Peptic ulcer disease, excluding bleeding	7.17	5.84		
Other neurological disorders	11.17	16.47		
Renal failure	36	34.8		
**Ontario myocardial infarction prediction rules [%]**				
Shock			1.55	1.48
Diabetes with complications			24.27	27.54
Congestive heart failure			34.3	35.36
Cancer			27.47	32.38
Cerebrovascular disease			31.6	38.15
Pulmonary edema			0.76	1.54
Acute renal failure			10.4	14.4
Chronic renal failure			20.8	27.5
Cardiac dysrhythmias			39	48.18

## Appendix A4: Regression results first-stage estimation

**Table:**

First-stage parameter estimates	Estimate	StErr		Prob Chi Sq
**Intervention characteristics**				
CABG fixed effect	9.4653		0.3084	< 0.0001
ICD/CRT fixed effect	9.2762		0.4887	< 0.0001
NSTEMI fixed effect	13.5200		501.0808	0.9785
STEMI fixed effect	13.3454		674.2201	0.9842
CABG readmission fixed effect	− 2.9075		0.2393	< 0.0001
ICD/CRT readmission fixed effect	− 2.9628		0.4960	< 0.0001
NSTEMI readmission fixed effect	− 6.9881		501.0808	0.9889
STEMI readmission fixed effect	− 7.3202		674.2201	0.9913
**Patient characteristics**				
Age	0.0112		0.0023	< 0.0001
Age × age	− 0.0001		0.0000	< 0.0001
Sex	− 0.0154		0.0077	0.0461
**Therapy**				
Anticoagulants Antiplatelet Anticoagulants × antiplatelet	− 0.1798 − 0.1195 − 0.0214		0.0116 0.0112 0.0145	< 0.0001 < 0.0001 0.1393
**Elixhauser comorbidity groups (CABG, ICD/CRT)**				
Blood loss anemia	− 0.2086		0.0267	< 0.0001
Hypertension, complicated	0.0310		0.0101	0.0022
Hypertension, uncomplicated	0.1488		0.0160	< 0.0001
Chronic pulmonary disease	0.0322		0.0098	0.0010
Depression	− 0.0144		0.0098	0.1434
Drug abuse	− 0.1857		0.0275	< 0.0001
Weight loss	− 0.0272		0.0169	0.1076
Cardiac arrhythmia	− 0.0256		0.0136	0.0597
Valvular disease	− 0.0016		0.0099	0.8723
Congestive heart failure	− 0.0245		0.0103	0.0178
Psychoses	− 0.1621		0.0249	< 0.0001
Fluid and electrolyte disorders	− 0.1195		0.0126	< 0.0001
Hypothyroidism	− 0.0048		0.0108	0.6546
Metastatic cancer	0.1296		0.0223	< 0.0001
Solid tumor without metastasis	0.0272		0.0114	0.0175
Obesity	− 0.0548		0.0101	< 0.0001
AIDS/HIV	0.0262		0.0967	0.7868
Alcohol abuse	0.0367		0.0185	0.0474
Rheumatoid arthritis/collagen	− 0.0204		0.0110	0.0642
Diabetes, complicated	− 0.0616		0.0116	< 0.0001
Diabetes, uncomplicated	0.0117		0.0131	0.3711
Peripheral vascular disorders	− 0.0054		0.0099	0.5846
Coagulopathy	− 0.0758		0.0117	< 0.0001
Liver disease	− 0.0195		0.0103	0.0579
Pulmonary circulation disorders	− 0.0656		0.0146	< 0.0001
Lymphoma	− 0.0459		0.0291	0.1150
Paralysis	− 0.1340		0.0168	< 0.0001
Deficiency anemia	− 0.0728		0.0125	< 0.0001
Peptic ulcer disease, excluding bleeding	− 0.0531		0.0182	0.0035
Other neurological disorders	− 0.0048		0.0129	0.7092
Renal failure	− 0.0790		0.0105	< 0.0001
**Ontario AMI prediction rules, distance (NSTEMI/STEMI)**				
Shock	− 0.0214		0.09	0.007
Diabetes with complications	0.0371		0.03	< 0.001
Congestive heart failure	− 0.1772		0.03	< 0.001
Cancer	0.0069		0.03	0.825
Cerebrovascular disease	− 0.0716		0.03	0.819
Pulmonary edema	− 0.2488		0.06	< 0.001
Acute renal failure	− 0.0911		0.03	< 0.001
Chronic renal failure	− 0.0879		0.03	< 0.001
Cardiac dysrhythmias	− 0.0727		0.02	< 0.001
Distance	− 0.0010		0.0005	0.0537
**Hospital characteristics**				
Teaching hospital Private for-profit hospital Private not-for-profit hospital Public hospital Beds Department admissions Department discharges Specialist doc Nurses Private for-profit hospital × beds Private not-for-profit × beds Public hospital × beds Teaching hospital × beds GRP inhabitant DE East DE West	0.0027 − 0.1558 − 0.0770 0.0223 − 0.0001 0.0000 − 0.0000 − 0.0003 0.0001 0.0002 0.0002 0.0000 − 0.0001 − 0.0000 − 0.1590 − 0.8882		0.0318 0.0900 0.0657 0.0629 0.0002 0.0000 0.0000 0.0002 0.0000 0.0002 0.0002 0.0002 0.0000 0.0000 0.1739 0.4112	0.9313 0.0836 0.2410 0.7228 0.4989 0.3115 0.2179 0.2081 0.0459 0.2326 0.2869 0.7963 0.0068 < 0.0001 0.3605 0.0308
Fixed effects for hospitals × interventions × outputs included (= 2,162 fixed effects)				

## Appendix A5: AFT regression results second-stage estimation

**Table:**

First-stage parameter estimates	Estimate		St. err.		Prob. Chi sq.	
**Intervention characteristics**						
CABG fixed effect		0.3562		0.0345		**< 0.0001**
ICD/CRT fixed effect		0.1698		0.0268		**< 0.0001**
NSTEMI fixed effect		0.5518		0.0300		**< 0.0001**
STEMI fixed effect		0.0000				
CABG readmission fixed effect		− 3.3210		0.0249		**< 0.0001**
ICD/CRT readmission fixed effect		− 3.0581		0.0147		**< 0.0001**
NSTEMI readmission fixed effect		− 3.5410		0.0244		**< 0.0001**
STEMI readmission fixed effect		− 2.9202		0.0206		**< 0.0001**
**Patient characteristics**						
Age		0.0125		0.0019		**< 0.0001**
Age × age		− 0.0001		0.0000		**< 0.0001**
Sex		− 0.0247		0.0062		**< 0.0001**
**Therapy** Anticoagulants Antiplatelet Anticoagulants × antiplatelet		− 0.1491 − 0.1014 − 0.0292		0.0094 0.0091 0.0117		**< 0.0001** **< 0.0001** **0.0126**
**Elixhauser comorbidity groups (CABG, ICD/CRT)**						
Blood loss anemia		− 0.2172		0.0214		**< 0.0001**
Hypertension, complicated		0.0317		0.0081		**< 0.0001**
Hypertension, uncomplicated		0.1519		0.0128		**< 0.0001**
Chronic pulmonary disease		0.0334		0.0078		**< 0.0001**
Depression		− 0.0126		0.0079		0.1096
Drug abuse		− 0.2003		0.0220		**< 0.0001**
Weight loss		− 0.0177		0.0136		0.1922
Cardiac arrhythmia		− 0.0194		0.0110		0.0779
Valvular disease		− 0.0078		0.0079		0.3253
Congestive heart failure		− 0.0157		0.0083		**0.0580**
Psychoses		− 0.1566		0.0199		**< 0.0001**
Fluid and electrolyte disorders		− 0.1256		0.0101		**< 0.0001**
Hypothyroidism		− 0.0087		0.0086		0.3145
Metastatic cancer		0.1121		0.0180		**< 0.0001**
Solid tumor without metastasis		0.0349		0.0092		**0.0001**
Obesity		− 0.0442		0.0081		**< 0.0001**
AIDS/HIV		0.0195		0.0779		0.8026
Alcohol abuse		0.0403		0.0148		**0.0066**
Rheumatoid arthritis/collagen		− 0.0218		0.0089		**0.0137**
Diabetes, complicated		− 0.0684		0.0093		**< 0.0001**
Diabetes, uncomplicated		0.0108		0.0105		0.3024
Peripheral vascular disorders		− 0.0034		0.0079		0.6640
Coagulopathy		− 0.0818		0.0093		**< 0.0001**
Liver disease		− 0.0265		0.0083		**0.0013**
Pulmonary circulation disorders		− 0.0847		0.0117		**< 0.0001**
Lymphoma		− 0.0387		0.0233		0.0974
Paralysis		− 0.1239		0.0134		**< 0.0001**
Deficiency anemia		− 0.0635		0.0100		**< 0.0001**
Peptic ulcer disease, excluding bleeding		− 0.0573		0.0145		**< 0.0001**
Other neurological disorders		− 0.0070		0.0104		0.5012
Renal failure		− 0.0888		0.0084		**< 0.0001**
**Ontario AMI prediction rules, distance (NSTEMI/STEMI)**						
Shock		0.1195		0.0398		**0.0027**
Diabetes with complications		− 0.0048		0.0112		0.6673
Congestive heart failure		− 0.1452		0.0108		**< 0.0001**
Cancer		0.0129		0.0111		0.2441
Cerebrovascular disease		− 0.0511		0.0106		**< 0.0001**
Pulmonary edema		− 0.2782		0.0464		**< 0.0001**
Acute renal failure		− 0.0787		0.0153		**< 0.0001**
Chronic renal failure		− 0.0584		0.0126		**< 0.0001**
Cardiac dysrhythmias		− 0.0176		0.0106		0.0960
Distance		− 0.0005		0.0004		0.2656
**Hospital characteristics**						
Teaching hospital		0.0336		0.0250		0.1797
Private for-profit hospital		− 0.1737		0.0710		**0.0144**
Private not-for-profit hospital		− 0.0206		0.0525		0.6949
Public hospital		0.0306		0.0503		0.5432
Beds		− 0.0000		0.0001		0.89
Department admissions		0.0000		0.0000		**< 0.0001**
Department discharges		− 0.0000		0.0000		0.7357
Specialist doc		− 0.0001		0.0002		0.4384
Nurses		0.0000		0.0000		0.5551
Private for-profit hospital × beds		0.0001		0.0001		0.438
Private not-for-profit private × beds		0.0001		0.0001		0.4136
Public hospital × beds		− 0.0000		0.0001		0.9105
Teaching hospital × beds		− 0.0001		0.0000		**0.0004**
GRP inhabitant		− 0.0000		0.0000		**< 0.0001**
DE East		− 0.1180		0.1409		0.4022
DE West		− 0.8525		0.3190		**0.0075**
452 hospital fixed effects included						

Note: The AFT model assumes that a covariate accelerates or decelerates the survival time by a constant. We applied a transformation for a better interpretation of coefficients’ magnitude [62]

### Case mix

Both age and age^2^ were significant (*p* < 0.0001) and presented an opposite effect on time to death and readmission. Each additional year was associated with a 1.2% increase in expected time to event. Conversely, for each unit of age^2^ we obtained a 1% decrease in time to death or readmission. Women (female = 1) resulted more exposed to shorter time to failure. For a female, the expected time to readmission or death was 2.5% shorter than for men (ODDS 0.975). All the combinations of pharmacotherapy (*p* < 0.0001) revealed a negative association with time to event: anticoagulant therapy alone and antiplatelet therapy alone had worse results for survival (− 14 and − 10%, respectively) than combination anticoagulant and antiplatelet therapy (− 3%).

The Ontario myocardial infarction (OMI) covariates were employed to assess the severity of conditions for STEMI and NSTEMI. The following variables met the 0.01 criterion for statistical significance: pulmonary edema (ODDS = 0.76) and heart failure (ODDS = 0.86) showed the greatest acceleration to death or readmission. Cerebrovascular disease (ODDS = 0.95), acute renal failure (ODDS = 0.92) and chronic renal failure (ODDS = 0.94) revealed a time to failure that was still negative but only slightly longer. Only shock had a positive coefficient (ODDS = 1.13).

The Elixhauser Index was applied to patients undergoing CABG or ICD/CRT. The significant coefficients associated with the shortest time to event were blood loss anemia (ODDS = 0.80), drug abuse (ODDS = 0.82), psychosis (ODDS = 0.86), electrolytes disorders (ODDS = 0.88) and paralysis (ODDS = 0.88). Obesity (ODDS = 0.96), rheumatoid arthritis (ODDS = 0.97), complicated diabetes (ODDS = 0.93), coagulopathy (ODDS = 0.92), liver disease (ODDS = 0.97), pulmonary circulation disorders (ODDS = 0.92), deficiency anemia (ODDS = 0.94), peptic ulcer disease (ODDS = 0.94), renal failure (ODDS = 0.91) contributed negatively to survival and time to readmission. Among the comorbidities with a positive coefficient and a longer time to failure, uncomplicated hypertension (ODDS = 1.16) and metastatic cancer (ODDS = 1.12) had the largest values. Chronic pulmonary disease (ODDS = 1.03), non-metastatic cancer (ODDS = 1.035), alcohol abuse (ODDS = 1.04) and complicated hypertension (ODDS = 1.03) increased time to event with a smaller effect [62].
